# Small molecule activators of the p53 response

**DOI:** 10.1093/jmcb/mjz006

**Published:** 2019-01-25

**Authors:** Marcus J G W Ladds, Sonia Laín

**Affiliations:** 1 Department of Microbiology, Tumor and Cell Biology, Biomedicum, Solnavägen 9, Karolinska Institutet, Stockholm, Sweden; 2 SciLifeLab, Tomtebodavägen 23A, Solna, Stockholm, Sweden

**Keywords:** cancer, p53, small molecule, cell biology, cancer therapy

## Abstract

Drugging the p53 pathway has been a goal for both academics and pharmaceutical companies since the designation of p53 as the ‘guardian of the genome’. Through growing understanding of p53 biology, we can see multiple routes for activation of both wild-type p53 function and restoration of mutant p53. In this review, we focus on small molecules that activate wild-type p53 and that do so in a non-genotoxic manner. In particular, we will describe potential approaches to targeting proteins that alter p53 stability and function through posttranslational modification, affect p53’s subcellular localization, or target RNA synthesis or the synthesis of ribonucleotides. The plethora of pathways for exploitation of p53, as well as the wide-ranging response to p53 activation, makes it an attractive target for anti-cancer therapy.

## Introduction

The tumour suppressor p53 in its role as the ‘guardian of the genome’ exerts a multitude of effects on cells in response to cellular stress (Lane, 1992). As a key transcription factor, it regulates a number of genes involved in cell cycle arrest, apoptosis, senescence, DNA repair, metabolism, autophagy, and ferroptosis (Wynford-Thomas, 1996; Gao et al., 2000; Ryan, 2011; Wang et al., 2016). As it plays such a central role in response to cellular stresses, the pathway is often dysregulated in cancer through either deletion or mutation of p53 itself, upregulation of its negative regulators, or dysfunction of its downstream effectors.

More than one half of solid tumours occurring in adults carry deletions or mutations in *TP53*, making it one of the most consistently and frequently mutated genes in cancer (Hollstein et al., 1991; Levine et al., 1991; Greenblatt et al., 1994). Whilst this is a significant number, it means that around 50% of solid tumours in adults carry wild type *TP53.* In addition, many haematological malignancies, tumours associated with viral infection, as well as childhood cancers, seldom carry *TP53* mutations. Wild type p53 status is very clearly associated with a positive clinical outcome and to susceptibility to chemoimmunotherapy in patients with chronic lymphocytic leukaemia (Malcikova et al., 2018) but this positive association is less clear in other cancer types. Tumours may possess wild type p53, but it may not be fully active and its response to stress may be dampened by alterations in other factors such as amplification of the p53 ubiquitin E3 ligase HDM2, loss of p14^ARF^ tumour suppressor or the expression of oncoviral proteins that inhibit p53 and target it for degradation (Kensuke and Vassilev, 2015). Therefore, promoting p53 expression and function in these tumours could have a beneficial effect for patients.

One salient point to consider is that classic chemotherapeutics and radiation therapy activate p53 by promoting its phosphorylation, which prevents p53 degradation, as well as, perhaps, also promoting p53 synthesis (Takagi et al., 2005; Chen and Kastan, 2010). In this review, we focus on the efforts made to achieve p53 activation in cancers using molecules that, in principle, do not cause damage to the genome, and are therefore less likely to cause irreversible side effects and treatment-related tumours. These strategies include molecules that activate p53 by modulating its posttranslational modifications, localization, synthesis and its degradation.

## Targeting posttranslational modifications of p53

**Figure 1:**
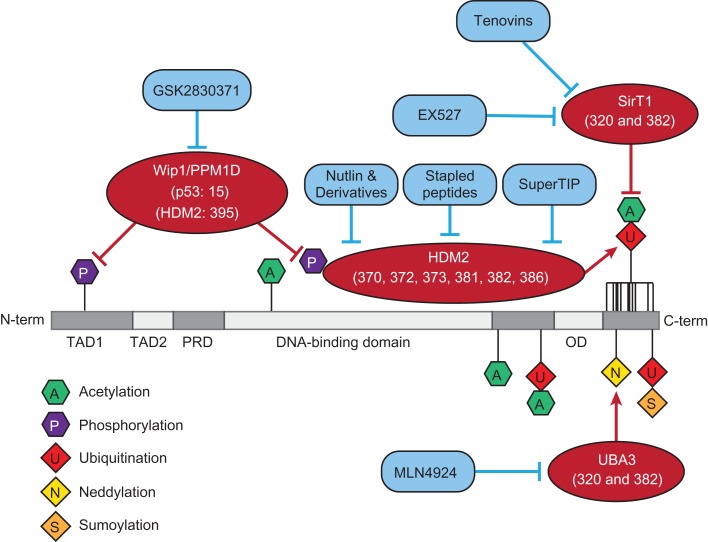
Summary of the key posttranslational modifications that are either deactivating (diamond) or activating (hexagon) and the causing proteins (red ovals). Inhibitors of each of the proteins are detailed in blue rectangles.

Finding small molecules that enhance or prevent modifications of p53 has long been considered a prospective strategy to treat tumours that retain wild type p53. Each of these strategies is summarized in Figure 1.

### Targeting HDM2/HDMX

The p53 protein itself is tightly controlled and maintained at low levels under normal cellular conditions by its primary negative regulator HDM2 (the human homologue of MDM2). HDM2 acts as an E3 ubiquitin ligase, which binds to p53 and subsequently causes its ubiquitination and nuclear export, targeting p53 for proteasomal degradation (Hock and Vousden, 2010). Interestingly, HDM2 participates in a negative feedback loop for p53 as it is also a target gene of p53. The induction of the tumour suppressor p14^ARF^ by oncogenes, and other stress signals, suppresses HDM2 through direct p14^ARF^ binding, providing a positive pressure during cellular stress for p53 activation (Sherr and Weber, 2000; Sherr, 2012). The HDM2 binder, HDMX/HDM4 — itself a structural homologue of HDM2, does not have any E3 ubiquitin ligase activity of its own. However, it does participate in the regulation of p53 through binding to its N-terminus as well as aiding HDM2 to increase ubiquitination of p53 by forming a heterodimer with HDM2 (Huang et al., 2011; Graves et al., 2012).

One of the first reagents published that targeted HDM2 was a 12 amino acid mini-protein derived from the HDM2 binding domain of p53 cloned into the active-site loop of *Escherichia coli* thioredoxin (Bottger et al., 1997). This protein insert was known as a thioredoxin insert protein (TIP), and the best of these, with an IC_50_ of 300 nM against HDM2, was named superTIP (Bottger et al., 1997). The development of superTIP paved the way for further development of peptide-based therapies including the use of stapled peptides. These peptides utilized a hydrocarbon bridge across the helical binding domain to stabilize its secondary structure, theoretically overcoming the limitation of the superTIP peptide sequence, which does not form a stable secondary structure (Bernal et al., 2007). The stapling of the α-helical portion of the p53 transactivation domain responsible for binding to the hydrophobic cleft of HDM2, stabilized the secondary structure and increased the potency of some peptides to yield a K_d_ as low as 6.76 nM (Brown et al., 2013). Stapled peptides are now reaching clinical trials, with one of the most advanced agents being ALRN-6924, an inhibitor of HDM2 and HDMX interaction with p53, having undergone phase I clinical trial (Meric-Bernstam et al., 2017). ALRN-6924 is currently at the time of writing undergoing further investigation for new phase I clinical trials either as a monotherapy or in combination with current therapeutics.

The first small molecule inhibitor identified as capable of disrupting the p53–HDM2 interaction was 4,5-dihydroimidazoline (nutlin) (Vassilev et al., 2004). The more potent compound, nutlin-3, possessed a chiral centre and demonstrated stereoselective inhibition of HDM2 with nutlin-3a being the active enantiomer. The nutlins spearheaded the development of more potent inhibitors including the chromenotriazolpyrimidine molecules, the terphenyls, the chalcones, and the benzodiazepinedione families (Orner et al., 2001; Go et al., 2005; Grasberger et al., 2005; Allen et al., 2009). Currently, nutlin-like molecules AMG232 (Sun et al., 2014) and idasanutlin (Ding et al., 2013) are both undergoing clinical trials (Table 1).

**Table 1:** Ongoing clinical trials of HDM2 inhibitors in combination with standard chemotherapeutics or radiation.

Compound	Drug combination	Tumour	Trial phase	Status	Trial identifier
Idasanutlin	Cytarabine	Acute myeloid leukaemia	III	Recruiting	NCT02545283
	None	Polycythemia vera	II	Recruiting	NCT03287245
	None	Solid tumours	I	Active not recruiting	NCT03362723
	None	Solid tumours	I	Completed	NCT02828930
	Ixazomib citrate and dexamethasone	Multiple myeloma	I and II	Recruiting	NCT02633059
	Pegasys	Polycythemia vera	I	Active not recruiting	NCT02407080
	Venetoclax and cobimetinib	Acute myeloid leukaemia	I and II	Recruiting	NCT02670044
	Obinutuzumab and rituximab	Follicular lymphoma	I and II	Active not recruiting	NCT02624986
	Atezolizumab and cobimetinib	ER^+^ Her2^–^ breast cancer	I and II	Recruiting	NCT03566485
	Rituximab, obinutuzumab, and venetoclax	Diffuse large B-cell lymphoma	I and II	Recruiting	NCT03135262
	Atezolizumab and cobimetinib	ER^+^ breast cancer	I and II	Recruiting	NCT03566485
	Radiation, alectinib, atezolizumab, vismodegib, temsirolimus, and palbociclib	Glioblastoma	I and II	Recruiting	NCT03158389
AMG232	None	Solid tumours or multiple myeloma	I	Completed	NCT01723020
	Radiation	Glioblastoma or gliosarcoma	I	Suspended	NCT03107780
	Trametinib and dabrafenib	Metastatic melanoma	Ib/IIa	Active not recruiting	NCT02110355
	Decitabine	Acute myeloid leukaemia	I	Recruiting	NCT03041688
	Trametinib	Acute myeloid leukaemia	Ib	Completed	NCT02016729
	Radiation	Soft tissue sarcoma	I	Recruiting	NCT03217266
DS-3032b	Cytarabine	Acute myeloid leukaemia	I and II	Not yet recruiting	NCT03634228
	None	Solid tumours and lymphomas	I	Recruiting	NCT01877382
	None	Acute myeloid leukaemia	I	Recruiting	NCT03671564
	None	Multiple myeloma	I	Recruiting	NCT02579824
	Quizartinib	FLT3-ITD mutant acute myeloid leukaemia	I	Recruiting	NCT03552029
	5-Azacytidine	Acute myeloid leukaemia, myelodysplastic syndrome	I	Recruiting	NCT02319369
HDM201	Trametinib	Colorectal cancer	I	Not yet recruiting	NCT03714958
	Anciliary treatment	Advanced solid and haematological *TP35* wt tumours	I	Active not recruiting	NCT02143635
	LCL161, everolimus, panobinostat, and QBM076	Colorectal cancer, non-small cell lung carcinoma, triple negative breast cancer, renal cell carcinoma	I	Recruiting	NCT02890069
	Cytarabine and anthracycline	Leukaemia (both myeloid and acute)	I and II	Not yet Recruiting	NCT03760445
	Ceritinib and/or trametinib	Neuroblastoma	I	Recruiting	NCT02780128
	LXS196	Uveal melanoma	I	Recruiting	NCT02601378
	Ribociclib (LEE011)	Liposarcoma	I and II	Active not recruiting	NCT02343172
APG115	Pembrolizumab	Metastatic melanoma or advanced solid tumours	I and II	Not yet recruiting	NCT03611868
	None	Solid tumours or lymphoma	I	Recruiting	NCT02935907
BI907828	None	Solid tumours	I	Recruiting	NCT03449381
ALRN-6924	None	Solid tumours and lymphomas	I and II	Recruiting	NCT02264613
	None/cytarabine	Acute myeloid leukaemia, myelodysplastic syndrome	I	Recruiting	NCT02909972
	None/cytarabine	Pediatric cancer	I	Recruiting	NCT03654716
	Paclitaxel	Solid tumours	I	Not yet recruiting	NCT03725436

### Targeting WIP1/PPM1D

p53 not only induces the expression of its E3 ligase HDM2 but also acts as a transcription factor for the expression of WIP1/PPM1D, another of its negative regulators. This p53 inducible phosphatase 1 not only destabilizes p53 by dephosphorylating serine 15 in p53 (Lu et al., 2005) but also dephosphorylates residue 395 in HDM2, which results in the stabilization of HDM2 (Lu et al., 2007). Furthermore, WIP1/PPM1D is either overexpressed, or mutated leading to increased activity in a variety of cancers expressing wild type p53 (Kleiblova et al., 2013). Thus, inhibitors of this enzyme have been considered as potential anticancer agents for wild-type p53 tumours. However, identifying small molecule inhibitors that have specificity for a particular phosphatase is challenging, and it was not until 2014 that a compound capable of specifically and potently inhibiting WIP1/PPM1D was identified (Gilmartin et al., 2014). This WIP1/PPM1D inhibitor, GSK2830371, can hinder tumour growth in a p53 dependent manner in lymphoma and a neuroblastoma model (Gilmartin et al., 2014; Chen et al., 2016). Although GSK2830371 is a weak inhibitor of proliferation on different cancer cell lines, it dramatically potentiates the effect of MDM2 inhibitors, especially in cells where there is a WIP1/PPM1D overexpression or hyperactivation (Esfandiari et al., 2016).

### Modulating p53 acetylation

The deacetylation of p53 at lysine residues has been implicated in enhancing its subsequent ubiquitination by HDM2 (Gu and Roeder, 1997; Tang et al., 2008). The class III histone deacetylase, SirT1, is one of the key players in p53 deacetylation (Luo et al., 2001). It has been shown that the combination of a SirT1 inhibitor, EX527, alongside DNA damage led to an increased acetylation of p53, though no increase in downstream p53 target genes (Solomon et al., 2006). Other SirT1 inhibitors such as the tenovins and inhauzin have been described to increase p53 levels and function (Lain et al., 2008; Zhang et al., 2012). However, at least in the case of the tenovins, the activation of p53 may be due to additional modes of action of these molecules as it has been shown previously that certain tenovins are capable of demonstrating target engagement with SirT1 without cells responding with a rise in levels or activity of p53 (Ladds et al., 2018a).

In 2002, it was reported that HDM2 is able to promote p53 deacetylation by recruiting a complex containing HDAC1, and therefore HDAC1 inhibition could promote p53 stability and function (Ito et al., 2002). Trichostatin A (TSA) is a potent inhibitor of class I and II mammalian histone deacetylases (HDAC), but not the class III HDACs such as the sirtuins. However, TSA on its own does not substantially increase levels of acetylated p53 (Sachweh et al., 2013). These results do not contradict previous studies on the potentiation of p53 acetylation by TSA, as these studies used cells that either overexpressed p300 acetyltransferase, or were irradiated with UV light, or treated with the DNA-damaging agent etoposide, for TSA to cause a detectable change p53 acetylation (Ito et al., 2001; Solomon et al., 2006). Altogether, these results suggest that in order for inhibitors of deacetylases to exert an effect on p53, acetylation of p53 must be induced prior to the addition of the deacetylase inhibitor.

### Targeting deubiquitinases

Another mechanism by which ubiquitination of p53 is altered is through the inhibition of deubiquitination. The deubiquitinase USP7 is responsible for the deubiquitination of both p53 and HDM2, though it displays a preferential activity towards HDM2 (Hu et al., 2006). A decrease in HDM2 ubiquitination leads to stabilization of HDM2, and subsequently to an increase in the ubiquitination and degradation of p53. USP7 has also been implicated in the deubiquitination of a number of key proteins involved in tumour suppression, such as PTEN (Song et al., 2008). In spite of the effects of USP7 inhibitors on multiple proteins, inhibitors of USP7 have been shown to be effective at raising p53 levels in cancer cells, and three such inhibitors, HBX 41108 (Colland et al., 2009), P22077 (Altun et al., 2011), and P5091 (Chauhan et al., 2012) led to p53-dependent effects on cell viability. Recently, two more potent inhibitors of USP7 have been identified, and one of them, FT671, has been shown to destabilize HDM2, increase levels of p53 and transcription of p53 target genes, and inhibit tumour growth in mice (Turnbull et al., 2017).

### Targeting NEDDylation

NEDDylation is another postranslational modification that can be modulated with small molecules. In the case of p53, NEDDylation is favoured by HDM2 and leads to inhibition of the transcription factor function of p53 (Xirodimas et al., 2004). FBX011 is another factor that promotes p53 NEDDylation and inactivation of p53 (Abida et al., 2007). It is, therefore, not a stretch to postulate that inhibition of NEDDylation may, in principle, activate p53. However, it is also possible that by inhibiting NEDDylation, ubiquitination of p53 may be favoured as both types of modification can occur at the same residues (Xirodimas et al., 2004). Indirect evidence suggests that NEDDylation may favour the detection of p53 in the nucleus, whereas ubiquitaned p53 is primarily detected in the cytoplasm (Brooks and Gu, 2006; Carter et al., 2007). Furthermore, NEDDylation occurs in two ribosomal proteins that modulate p53 degradation, RPL11 and RPS14 (Sundqvist et al., 2009; Zhang et al., 2014). In particular, NEDDylation of RPL11, which is promoted by MDM2, contributes to the nucleolar localization of RPL11, which could be associated with a loss in p53 stabilization. HDM2 also favours NEDDylation of RPS14 as well as being required for its nucleolar localization (Zhang et al., 2014).

MLN4924 (Pevonedistat) (Soucy et al., 2009) is thought to act as an AMP mimetic that forms a covalent adduct with NEDD8 in the catalytic pocket of the NEDD8-activating enzyme component UBA3. This compound has been tested in numerous clinical trials and in combination with 5-azacytidine. Additionally, phase III trials for myelodysplastic syndromes, chronic myelomonocytic leukaemia, or low-blast acute myelogenous leukaemia are recruiting at the time of writing. As with many small molecules targeting mechanisms that affect multiple cellular factors, the biological effect of MLN4924 can occur in the absence of p53 (Soucy et al., 2009). Despite this, it is clear that cellular context, including p53 status, can affect the type response to NEDD8 inhibition (Lin et al., 2010; Blank et al., 2013). For example, p53 knockdown can promote the apoptotic response to MLN4924 treatment in a breast cancer cell line (Lin et al., 2010). In a related study, it was a shown that cytostatic activation of p53 with a low-dose of actinomycin D can lead to protection of cancer cells from MLN4924 (Malhab et al., 2016).

### Targeting the proteasome

Inhibitors of the proteasome, including the clinically approved bortezomib, rapidly increase p53 protein levels (Williams and McConkey, 2003). Not only is the degradation of p53 inhibited, its localization also remains nuclear upon bortezomib treatment (Williams and McConkey, 2003). Combination of bortezomib with the HDM2 antagonist, nutlin-3, led to synergistic cell kill in a number of different tumour types with wild-type p53 (Ooi et al., 2009). It should be noted that in p53 mutant or null cells, bortezomib has recently been found to act to induce apoptosis through the p53 homologue, p73 (Dabiri et al., 2017).

## Inhibition of p53 nuclear export

For p53 to exert its transcription factor function, it requires nuclear localization. HDM2-mediated ubiquitination of p53 causes its nuclear export and subsequent proteasomal degradation (Geyer et al., 2000). Therefore, one of the possible mechanisms to promote p53 transcription factor function and prevent its degradation may be to block its nuclear export. Leptomycin B was found to be a inhibitor of CRM1, a nuclear export protein (Nishi et al., 1994). Leptomycin B covalently inhibits CRM1 through a Michael addition to a cysteine residue on CRM1 (Kudo et al., 1999), a feature that may explain the potency of this natural compound. Accordingly, leptomycin B is capable of increasing p53 levels and its nuclear localization and do so at very low concentrations (Freedman and Levine, 1998; Lain et al., 1999; Smart et al., 1999; Hietanen et al., 2000; Menendez et al., 2003). However, the use of leptomycin B in the clinic was limited due to its toxicity. New inhibitors of nuclear export have been identified that possess the potency of leptomycin B, but apparently lack the same toxicities (Mutka et al., 2009). Selinexor (KPT-330) is another CRM1 inhibitor currently being evaluated following phase II trials as well as further recruitment of participants for phase III clinical trials for the treatment of various cancers. KPT-8602 is yet another CRM1 blocker being evaluated being evaluated as an anti-cancer agent in a phase I clinical trial (NCT02649790) (Etchin et al., 2017).

## Activation of p53 through depletion of ribonucleotides

Indirect activation of wild-type p53 by depleting cells of ribonucleotides is another possible strategy to activate p53 without causing DNA damage (Linke et al., 1996). In the past couple of years, targeting of dihydroorotate dehydrogenase (DHODH), a key enzyme in the *de novo* pyrimidine synthesis pathway, has gained increasing attention following a seminal paper by Sykes et al. (2016) demonstrating that acute myeloid leukaemia (AML) cells could undergo differentiation upon DHODH inhibition. A link between DHODH and p53 induction has been suggested since 2010 in HeLa cells, where p53 degradation is mediated by the action of the E6 viral oncoprotein (Khutornenko et al., 2010). However, it is only recently that it was found that DHODH inhibition results in the increase in p53 at early timepoints in a cancer cell line where p53 degradation is mediated by MDM2 (Ladds et al., 2018b). At least part of the induction of p53 in these cells is due to an increase in synthesis. p53 mRNA is relatively unchanged by DHODH inhibitors, meaning that the rise in p53 protein is most likely due to an increase in translation of the mRNA already present. The p53 transcribed appears to be functionally active and able to induce the transcription of p53 target mRNAs. In cells with defective cell-cycle checkpoints, like many tumours, DHODH inhibition led to the accumulation of cells in S-phase and this associated with a lowering of levels of cdc6, a key factor involved in licencing of origins of replication (Blow and Hodgson, 2002; Evrin et al., 2009). It was also shown that DHODH inhibitor treated cells proceed into S-phase with a raised level of p53, a situation that may shift cells towards the death pathway. This is in contrast to the response seen by treating the same cells with nutlin-3 as the cells exhibit a classic p53-induced growth arrest in G1 and G2/M. DHODH inhibitors have been previously shown to be highly effective against various cancers (Hail et al., 2010; O’Donnell et al., 2012; Sykes et al., 2016; Ladds et al., 2018b), and one of the most potent inhibitors, brequinar, even made it to clinical trials in the early 1990s for the treatment of a variety of solid tumours, though it was judged to be too toxic to patients in light of its modest clinical outcomes (Dexter et al., 1985; Arteaga et al., 1989; Natale et al., 1992; Urba et al., 1992; Cody et al., 1993; Maroun et al., 1993; Moore et al., 1993).

In the paper by Sykes et al. (2016), this failure in clinical trials was suggested to be due to the inability of DHODH inhibitors to stop the growth of solid tumours in man and that better outcomes could be expected in the treatment of leukaemias. In this regard, Bayer has commenced a clinical trial (NCT03404726) with a novel and very potent DHODH inhibitor, BAY2402234. One possible way to exploit DHODH inhibition and minimize any on-target toxicities that DHODH inhibition may have *in vivo* would be to combine with a relevant therapy. Indeed, we have already shown that combination of a DHODH inhibitor, HZ00/HZ05, with an inhibitor of p53 degradation, nutlin-3, led to a synergistic tumour cell kill both *in vitro* and *in vivo* in tumour xenograft studies (Ladds et al., 2018b).

It is not only DHODH within the *de novo* pyrimidine pathway that has been tested in clinical trials, but also the trifunctional multi-domain enzyme CAD, the enzyme responsible for the first three steps of the *de novo* pathway. The compound, N-(phosphonacetyl)-L-aspartate (PALA) has been noted for its anti-tumour activity for many years having been tested in both *in vivo* tumour models as well as in patients (Johnson et al., 1976; Linke et al., 1996). It was found to be an inhibitor of CAD, exerting its activity on cells through that pathway (Collins and Stark, 1971). Cells with functional p53 underwent cell cycle arrest, predominantly in G1, whereas p53 deficient cells progressed through to S-phase and accumulated there (Linke et al., 1996). When further testing was conducted, it was found resistance to PALA was associated with the overexpression of CAD (Chen et al., 2001). The amplification of CAD was found to correlate with a reduction or deficiency in MLH1 or MSH6, two key mismatch repair enzymes (Chen et al., 2001). What is highly interesting is that in a recent article highlights the importance of MLH1 as a key contributor of p53-mediated tumour suppression (Janic et al., 2018). The concomitant upregulation of CAD with loss of a key effector of p53-mediated tumour suppression leading to a loss of the efficaciousness of PALA is an interesting avenue to explore for targeting the pyrimidine ribonucleotide synthetic pathway.

## Nucleolar stress

Most classic cancer therapeutics, through one means or another, are actually indirectly p53 activators (Weinstein et al., 1997). Many of these compounds, as well as other compounds that activate p53 but are not yet used in the clinic, cause disruption of nucleoli (Rubbi and Milner, 2003). In a model proposed by the discoverers of this association (Vlatkovic et al., 2014), the nucleolus would be an important site for p53 polyubiquitination and is, therefore, required for degradation of p53.

An archetypal example of a classic cancer therapeutic that causes nucleolar disruption as well as p53 activation is actinomycin D, which at low doses does not lead to detectable levels of DNA damage indicators (Choong et al., 2009). In this case, there is evidence suggesting that inhibition of rDNA transcription by actinomycin D can increase the interaction of MDM2 with free ribosomal proteins, and result in the blockage of MDM2-mediated p53 degradation (Golomb et al., 2014). In view of the results using actinomycin D, it is reasonable to expect that impairment of RNA polymerase function would stabilize and activate p53. Indeed, several RNA polymerase I inhibitors, such as CX5461, demonstrate p53 activation and p53 dependency for their effects on cell viability (Bywater et al., 2012). Other agents that disrupt nucleolar structure are the CX-3543, a small molecule capable of disrupting nucleolin/rDNA G-quadruplex complexes in the nucleolus (Drygin et al., 2009) for which two clinical trials have been completed though the results are, as of yet, not reported, and BMH-21, which induces degradation of RNA polymerase I (Peltonen et al., 2014; Wei et al., 2018).

Interestingly, by using a phenotypic screen for p53 transcription factor function, a high number of tubulin poisons were uncovered to be hits in the screen (Staples et al., 2008). How tubulin poisons increase p53 levels and transcription factor activity is not clear. One contributing mechanism could be related to the appearance of multinucleated cells upon disruption of the mitotic spindle. This disruption causes p53 accumulation in some of the nuclei of the same cell, but an absence in others (Staples et al., 2008). Based on the role for nucleoli in p53 degradation, we propose that p53 will only accumulate in the nuclei that do not contain a nucleolus.

## Inhibition of cyclin-dependent kinases

Inhibitors of certain cyclin-dependent kinases (CDKs) are also potent activators of the p53 response. For example R-roscovitine (Seliciclib), which targets multiple CDKs in the nanomolar range, induces the expression of functional p53 in cells (MacCallum et al., 2005). According to the current model, roscovitine may activate p53 by inhibiting HDM2 expression (MacCallum et al., 2005), a feature shared with another broad inhibitor of CDKs, flavopiridol. 5,6-dichloro-1-β-D-ribofuranosylbenzimidazole (DRB), another p53-activating compound also leads to a decrease in MDM2 levels. Because DRB is described as an inhibitor of RNA polymerase II (Yankulov et al., 1995), it can be speculated that the effects of roscovitine and flavopiridol on HDM2 may result from inhibition of the activity of CDK7/9 on the carboxy terminal domain of the large subunit of RNA polymerase II. However, the possibility of these compounds exerting effects though other mechanisms cannot be discarded. Strengthening this assertion was the observation that these compounds cause nucleoli disruption (Parker et al., 1998; Sirri et al., 2002; Rubbi and Milner, 2003). Regardless of the mechanism responsible for the activation of p53, the premise that CDK7/9 inhibition leads to activation of p53 is supported by studies using a new small molecule targeting CKIα as well as CDK7/9, which has recently shown to cure AML in mice through a mechanism that involves p53 activation (Minzel et al., 2018). It is worth remembering that in a previous study it was shown that CDK7 not only phosphorylates the RNA polymerase II C-terminal domain, but also the C-terminal residues in p53 and potentially improves the binding of p53 to DNA (Lu et al., 1997).

The inhibition of cell cycle progression through inhibition of CDK4 and CDK1 has been associated with the p53/HDM2 axis. *CDK4* and *HDM2* amplification are frequently linked in liposarcoma and the combination of inhibitors to each of these enzymes slightly improves the *in vivo* antitumor effect (Laroche-Clary et al., 2017). Further observations are required to consider this combination as an effective strategy. With regards to CDK1, it was reported that inhibition of MDM2 with nutlin and blockage of iASPP phosphorylation using a cyclin B1/CDK1 inhibitor activated p53 in melanoma cells (Lu et al., 2013). Furthermore, this reactivation of p53 cooperated with vemurafenib, a BRAF^V600E^ and BRAF^V600K^ inhibitor, to suppress melanoma growth *in vivo*, by inducing p53-dependent apoptosis and growth suppression.

## Conclusions

**Figure 2:**
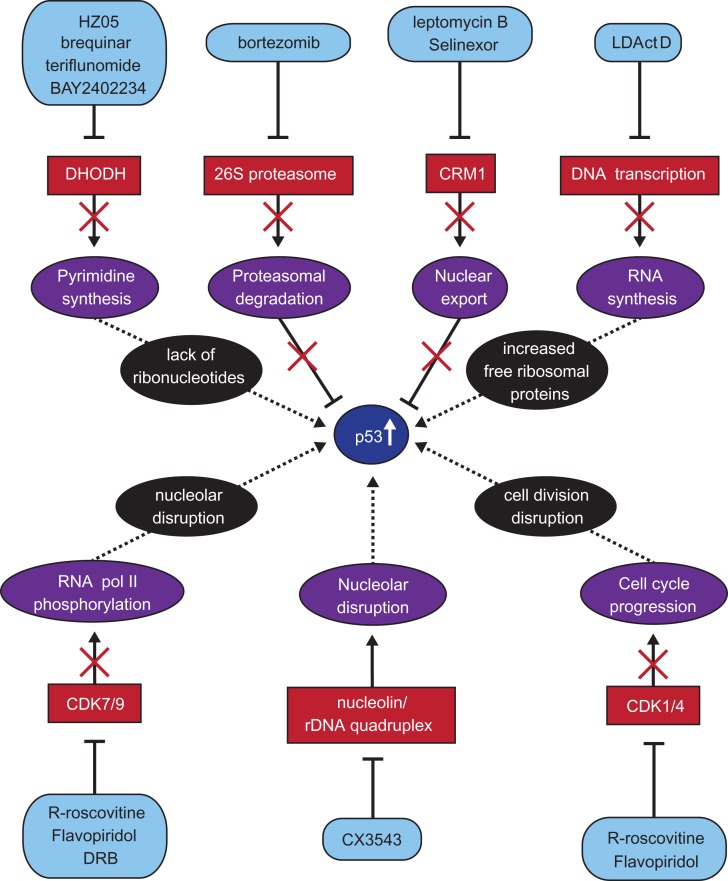
Summary of the potential p53 activation routes and the compounds involved.

The exploitation of the p53 pathway therapeutically has been a formidable challenge, and one that has been under investigation for more than two decades. As summarized in Figure 2, there are a plethora of possible routes to the same end of activating p53. Of the agents described above, the class of compounds that do not exhibit any effects on cell viability in p53 deficient cells are the HDM2 inhibitors. The compounds, however, that target proteins that exert effects on cells beyond the p53 pathway do exhibit an effect on p53 deficient cells although, in many instances, the type of outcome, such as the stage at which cells arrest in the cell cycle or whether induction of cell death occurs, does depend on the p53 status. It is also frequently seen that MDM2 inhibitors only results in arrest in G1 as well as in G2, and this stalling of the cell cycle is reversible upon removal of the compound. One consequence of this reversibility is limited the efficacy of compounds, furthermore, this may also increase the risk for endoreduplication and therefore genomic instability (Shen and Maki, 2010). Moreover, HDM2 inhibitors have been shown to exhibit on-target clinical toxicity (Khoo et al., 2014). Perhaps targeting HDMX as well as HDM2, as demonstrated for the stapled peptides, is a way forward (Chee et al., 2017).

Multiple clinical trials where HDM2 inhibitors are combined with standard chemotherapeutics or radiation are ongoing (Table 1). Studies on the combination of HDM2 inhibitors with targeted compounds that also activate the p53 pathway through mechanisms other than HDM2 inhibition, may constitute a strategy to achieve more effective cancer treatments, but these are still scarce. With regards to immunotherapy, it has been recently found that HDM2 family gene amplifications correlate with hyperprogression, that is accelerated tumour growth upon administration of immune checkpoint inhibitors (Kato et al., 2017). Therefore, it is tempting to speculate that inhibitors of HDM2 could have beneficial effects on these patients.

Finally, one must bear in mind that HDM2 inhibitors can protect normal cells from S-phase poisons, mitotic poisons, as well as other agents such as DHODH inhibitors. Therefore, the order in which these agents are administered could be postulated to be of great importance to mitigate an undesirable outcome and to be successful as treatments.
